# Cyclic Polyhydroxy Ketones II. *xylo*-Trihydroxycyclohexenediolic Acid and Keto-Inositols

**DOI:** 10.6028/jres.068A.026

**Published:** 1964-06-01

**Authors:** Alexander J. Fatiadi, Horace S. Isbell

## Abstract

A new crystalline compound, dl-*xylo*-trihydroxycyclohexenediolic acid (dl-*xylo*-pentahydroxy-2-cyelohexen-1-one) (I), has been isolated from the products of oxidation of *myo*-inositol with nitric acid, and its structure has been established. Compound (I) reduces Tillmans reagent, reacts with iodine in neutral or slightly acidic solution, produces a blue color with ferric chloride solution, and exhibits other properties characteristic of an enediolic acid. On catalytic reduction, it gives both *scyllo*-inositol and *myo*-inositol. On oxidation, it yields a new triketo-inositol, *xylo*-4,5,6-trihydroxycyclohexane-l,2,3-trione (II).

Under acidic conditions, catalytic acetylation of I gives two pentaacetates, the infrared spectra of which are similar but not identical. One of these acetates exists in two forms, both of which, on deacetylation, yield the parent acid I. The product formed by deacetylation of the other pentaacetate has not been identified. Benzoylation of I gives a crystalline pentabenzoate.

Under basic conditions, acetylation of I proceeds with simultaneous aromatization, resulting in the formation of pentaacetoxybenzene, from which pentahydroxybenzene is obtained by hydrolysis.

*xylo*-4,5,6-Trihydroxycyclohexane-l,2,3-trione (II) gives a crystalline bis(phenylhydrazone). By acetylation under basic conditions, it yields hexaacetoxybenzene.

Mechanisms are presented for the aromatization of keto-inositols by enolization and *beta* elimination-reactions, and certain observations reported in the literature are rationalized. Infrared and ultraviolet absorption spectra are reported for the new compounds.

## 1. Introduction

Cyclic polyhydroxy ketones, obtained by the oxidation of inositols, yield enolic compounds of value in the study of oxidation–reduction phenomena and molecular structure. Prior workers have established that oxidation of *myo*-inositol (III) with nitric acid gives dl-*epi*-inosose-2 (V), tetrahydroxy-*p*-benzoquinone, rhodizonic acid, and a residue weighing approximately half the weight of the parent *myo*-inositol [1 to 5].^3^ It has now been found that, after treatment with potassium carbonate, this residue gives a crystalline potassium salt of a new enediolic acid (I)^4^ in a yield of 10 percent (based on the weight of *myo*-inositol taken). On oxidation, the salt gives a new crystalline trihydroxycyclohexanetrione (II). The structures and properties of I and II have been investigated, and certain characteristic derivatives have been prepared.

## 2. dl-*xylo*-4,5,6-Trihydroxycy clohexenediolic Acid (I)

The structure of (I) was established from the following considerations: The equivalent weight and chemical analysis of the acid correspond to the formula C_6_H_8_O_6_; analysis of the potassium salt of the acid is in agreement with the formula C_6_H_7_KO_6_·H_2_O; both the acid and the salt react with two equivalents of either iodine or Tillmans reagent and give a deep-blue color on admixture with methanolic ferric chloride.

**Figure 1:**
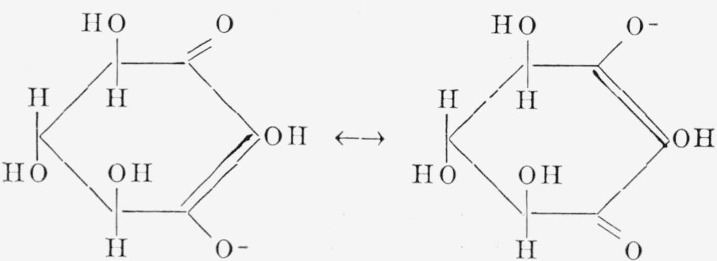
Anion of *xylo*-4, 5, 6-trihydroxycyclohexenediolic acid.

A compound having structure I may arise by enolization of either an *ortho*- or a *meta*-diketo-inositol.^5^ Compound I might have any one of several configurations: the actual configuration was established by studying the reduction products. On catalytic reduction with hydrogen, I gave a mixture of *myo*-inositol (III) and *scyllo*-inositol (IV). The latter has an alternating arrangement of the hydroxyl groups around the ring ([9], p. 138; [10]). Hence, the new enediolic acid (I) likewise must have an alternating arrangement of its three hydroxyl groups and must be dl-*xylo*-triliydroxycyclohexenediolic acid. In nonionic derivatives formed from the acid, the configuration is d-and l-*xylo* (racemic, unless diastereomers are formed). However, in the anion (fig. 1), Cl and C3 are identical because of resonance, and the configuration is *meso.*

The potassium salt is particularly suitable for isolating and purifying the compound.

**Figure 2:**
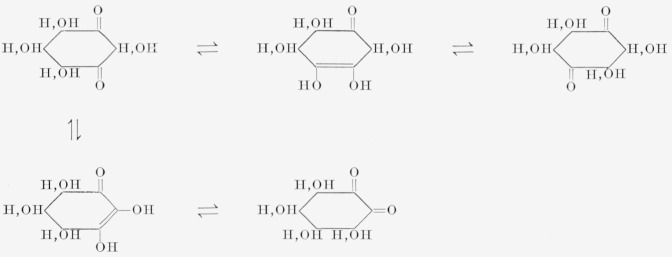
Inter conversion of diketo-inositols by enolization.

The isolation of I does not establish the structure of the parent diketo-inositol. Thus, as depicted in figure 2, all of the diketo-inositols are theoretically interconvertible by reversible enolizations, to yield a mixture of isomeric trihydroxycyclohexenediolic acids. Compound I is the only isomer thus far isolated.

Acetylation of I, catalyzed by mild acidic catalysts, gave a pentaacetate, mp 111 to 113 °C, which solidified above 113° and then remelted at 134 to 136 °C. Recrystallization of the higher-melting acetate from ethanol produced the lower melting. The infrared spectra of the two compounds are identical, and hence they are polymorphic modifications of the same compound. Deacetylation of both modifications regenerated I; therefore, the acetylation had not altered the configuration, and the compound is dl-*xylo*-pentaacetoxy-2-cyclohexen-1-one. Benzoylation of I, with zinc chloride as catalyst, gave a pentabenzoate shown to be dl-*xylo*-pentabenzoyloxy-2-cyclo hexen-1-one.

Acetylation of I, catalyzed by sulfuric acid, gave a pentaacetate (mp 154 to 155 °C) presumably having an altered configuration. The substance differs from that described above in its melting point, in its infrared spectrum, and in the fact that, on deacetylation, it does not yield I. However, it has essentially the same ultraviolet spectrum.

**Figure 3:**
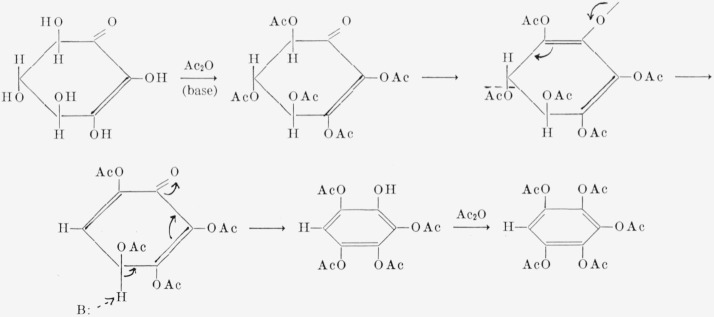
Mechanism for the formation of pentaacetoxybenzene from I.

Acetylation of I under basic conditions gave pentaacetoxybenzene, presumably by the series of enolization and *beta*-elimination reactions depicted in figure 3 [11, 12]. On deacetylation, the pentaacetate gave pentahydroxybenzene, an important substance neither generally available nor closely investigated [13, 14].

## 3. *xylo*-4,5,6-Trihydroxycyclohexane-1,2,3-trione (II)

When oxidized with concentrated nitric acid, the new enediolic acid (I) gave a crystalline product (II). The analysis, molecular weight, and other properties of the latter show that it is *xylo*-4,5,6-trihydroxycyclohexane-1,2,3-trione. Crystalline II, the first triketoinositol to be isolated, provides a promising new area for investigation. The relationship of I to II is the same as that of ascorbic acid to dehydroascorbic acid.

**Figure 4:**
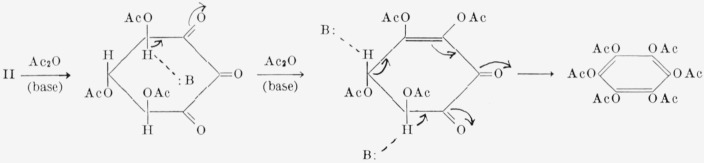
Mechanism for the formation of hexaacetoxybenzene from II.

Acetylation of II under basic conditions gave hexaacetoxybenzene [15, 16, 17], presumably by the process depicted in figure 4. Compound II yielded a crystalline bis(phenylhydrazone) whose structure is being investigated.

## 4. Mechanisms for Aromatization of Keto-Inositols [11, 12]

**Figure 5:**
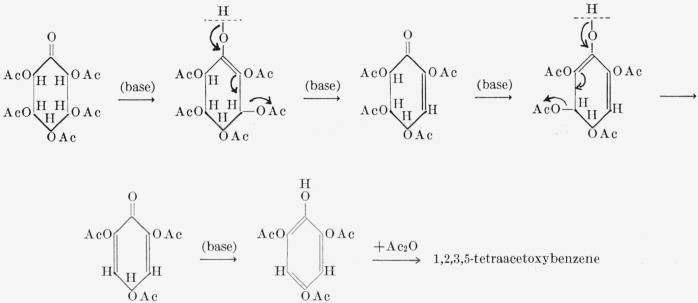
Mechanism for the formation of 2-hydroxy-1,3,5-triacetoxybenzene from inososes by acetylation [11, 12].

For many years, inositols have been considered to be possible precursors of certain naturally occurring, aromatic compounds. In 1936, Posternak [3] showed that, following treatment with sodium acetate and acetic anhydride, or with pyridine and acetic anhydride, the pentaacetates and pentabenzoates of dl-*epi*-inosose-2 (V) and *myo*-inosose-2 (VI) yield 1,2,3,5-tetrahydroxybenzene. Under milder conditions, the pentabenzoate of *myo*-inosose-2 gives 1-hydroxy-2,3,5-tribenzoyloxybenzene (2,3,5-tribenzoyloxyphenol). Posternak [4] suggested that the aromatization of the inosose takes place through an enolic intermediate. Isbell [11, 12] depicted the process as involving successive enolization reactions with cleavage of acyloxy groups *beta* to the enolic hydroxyl groups (fig. 5). He predicted that the reaction would lead to 2-hydroxy-1,3,5-tribenzoyloxybenzene (2,4,6-tribenzoyloxyphenol) instead of the 1-hydroxy-2,3,5-tribenzoyloxybenzene actually found. In 1959, Angyal and Anderson ([9], p. 179) reported that heating penta-*O*-acetyl-*myo*-inosose-2 in soft glass yields 2,4,5,6-tetraacetoxy-2-cyclohexen-1-one (VII), one of the intermediates postulated by Isbell. Later, Stanacev and Kates [18] isolated VII by treating the acetates of *myo*-inosose-2 and dl-*epi*-inosose-2 with platinum oxide and hydrogen. These authors accounted for a number of discrepancies in the literature by showing that the pentaacetates and pentabenzoates of inososes are easily altered by alkali-catalyzed reactions. Posternak and Peshusses [19] also isolated VII. They suggested that the 1-hydroxy-2,3,5-tribenzoyloxybenzene previously reported arises by migration of a benzoyl group from the oxygen of Cl to that of C2 by a neighboring-group mechanism. Thus, Isbell’s postulated mechanism for the aromatization has largely been confirmed, and the unexpected formation of the 1-hydroxy-2,3,5-tribenzoyloxybenzene has now been explained.

## 5. Experimental Procedures

### 5.1. Oxidation of *myo*-Inositol With Nitric Acid, and Separation of Products

Commercial *myo*-inositol (100 g) was added, with stirring, to 300 ml of 5-*N* nitric acid^6^ in a large evaporating dish, and the stirred mixture was heated to boiling. Heating and stirring were continued until a sample of the material taken on a glass rod was a milky-white, viscous sirup; this required about 70 min. During the final stage, the mixture was heated cautiously, to avoid charring. The mixture was placed over moistened sodium hydroxide in a desiccator, and concentrated under reduced pressure to a semisolid, foamy residue (about 60 min) which was triturated with 90 ml of hot water and kept in a refrigerator for 24 hr. The resulting crude, crystalline dl-*epi*-inosose-2 was collected by filtration and washed with ice water; wt, 10 g (air-dried). A sample of the inosose, purified through its phenylhydrazone by the method of Posternak [20], melted at 206 to 208 °C dec.

The mother liquor (about 150 ml) was stirred and gradually brought to *p*H 7 to 7.5 by addition of potassium carbonate (40 to 43 g), heated for 15 min on a boiling water-bath, allowed to stand at room temperature for 15 min, and kept in ice for 45 min, with occasional stirring. The resulting dark, greenish-blue crystals were collected on a filter, successively washed with ice water and cold, 50-percent aqueous methanol, and air-dried; wt, 15 to 18 g. The crude product contained approximately equal quantities of the dipotassium salts of rhodizonic acid and tetrahydroxy-*p*-benzoquinone.^7^

The mother liquor was concentrated at 40 °C under reduced pressure to about 100 ml, at which point crystallization began; the mixture was stored overnight in a refrigerator. The resulting prismatic crystals were collected by filtration (polyethylene dam) and successively washed with ice water, 50-percent methanol, and anhydrous methanol. A small second crop was obtained by concentrating and cooling the filtrate. The combined, crude, light-yellow product was dried; wt, 25 g. By analysis, it contained about 75 percent of the potassium salt of I.

For purification, the crude salt was dissolved in 300 ml of freshly boiled, distilled water at 55 °C. The solution was treated with 5 g of acid-washed, activated carbon,^8^ the suspension was filtered, and the residue was washed with freshly boiled water. The combined, light-brown filtrate and washings were saturated with acetone (about 500 ml) and gradually cooled. The resulting, reddish-orange crystals^9^ were collected by filtration, washed successively with cold, 75-percent aqueous acetone and anhydrous acetone, and dried; wt, 17.5 g. By analysis, they contained 98 percent of potassium trihydroxycyclohexenediolate monohydrate.

From the residue, after separation of the pot assium salt of I, the following were isolated through their copper complexes: compound I (small proportion) ; rhodizonic acid; tetrahydroxy-*p*-benzoquinone; and croconic acid. Oxalic acid was separated from the original residue as the bis(phenylhydrazide).

The potassium salt of I was also obtained by precipitation of the oxidation products (after separation of dl-*epi*-inosose-2) with excess glacial acetic acid. On neutralizing the resulting colorless, amorphous products with potassium carbonate and treating the solution as described above, the potassium salts of tetrahydroxybenzoquinone, rhodizonic acid, and the enediolie acid (I) were crystallized in approximately the yields reported above.

### 5.2. DL-*xylo*-Trihydroxycyclohexenediolic Acid (I)

#### a. Preparation and Properties

A solution of 35 g of the recrystallized potassium salt of I in 650 ml of freshly boiled water was passed through 500 ml of a cation-exchange resin (Amberlite IR120–H) into 200 ml of 50-percent, aqueous acetic acid. The resin was washed with 1 liter of freshly boiled water. The combined effluent and washings were concentrated under reduced pressure to about 850 ml, treated with 25 g of acid-washed decolorizing carbon, and filtered. The colorless filtrate was mixed with 50 ml of acetic acid and concentrated under reduced pressure to about 400 ml, whereupon crystallization commenced. The mixture was diluted with 50 ml of acetic acid and stored overnight in a refrigerator; the resulting lustrous crystals were separated by filtration, washed with 50-percent, aqueous acetic acid, and dried in a vacuum desiccator over sodium hydroxide. The mother liquor, after concentration and dilution with acetic acid, gave a second crop of I; total wt, 18 to 20 g; mp, 210 to 211 °C dec., with darkening at 205 °C.

The crude product was dissolved in 600 ml of 10-percent aqueous acetic acid at 60 °C, 60 ml of glacial acetic acid and 15 g of acid-washed decolorizing carbon were added, and the suspension was filtered. The colorless filtrate was concentrated under reduced pressure to about 450 ml, whereupon crystallization began. The mixture was diluted with 50 ml of acetic acid, kept overnight in a refrigerator, and filtered. The crystals were washed with 50-percent, aqueous acetic acid, and dried; wt, 15 to 18 g; mp, 211 to 212 °C dec.

*Anal.* Calcd, for C_6_H_8_O_6_: C, 40.9; H, 4.6. Found: C, 41.0; H, 4.5.

The enediolic acid was also obtained by treating 10 g of the potassium salt with 50 g of warm, glacial acetic acid. After several hours, the difficultly soluble acid was separated by filtration and recrystallized from aqueous acetic acid; wt, 5 to 6 g.

A 74.1-mg sample of I required 4.20 ml of 0.1-*N* sodium hydroxide for neutralization; hence, the equivalent weight is 176, in agreement with the calculated molecular weight.

Furthermore, an 84.4-mg sample of I reacted with 9.57 ml of 0.1-*N* iodine, in agreement with an equivalent weight of 88.0 and a molecular weight of 176.

The enediolic acid reduces Benedict solution, silver nitrate, and periodic acid, gives a transitory blue color with methanolic ferric chloride, reacts with an equimolecular proportion of Tillmans reagent and of iodine, and forms an insoluble copper complex when added to a solution of cupric acetate ([6], p. 16).

#### b. Potassium Salt

Purified I (1 g) was dissolved at room temperature in 150 ml of freshly boiled water. The solution was neutralized under nitrogen with aqueous potassium hydroxide, cooled in an ice bath, and treated with cold acetone (about 300 ml) to incipient turbidity. The resulting colorless crystals were separated under nitrogen, washed with acetone, and dried over phosphorus pentoxide in a vacuum desiccator; the yield was nearly quantitative.

*Anal.* Caled, for C_6_H_7_KO_6_·H_2_O: C, 31.0; H, 3.9; K, 16.8. Found: C, 30.9; H, 3.9; K, 17.3.

A sample (108.1 mg) of the potassium salt reacted with 9.29 ml of 0.1-*N* iodine, in agreement with a molecular weight of 232.7 (calcd. M.W. 232.2). An aqueous solution of the salt gave a stable, deep-blue color with methanolic ferric chloride.

When the purified potassium salt of I was heated with 1-*N* potassium hydroxide and active manganese dioxide by the procedure of reference [17], croconic acid was isolated (as the barium salt) in a yield of about 10 percent.

#### c. Ammonium Salt

A solution of 200 mg of I in 10 ml of freshly boiled water at 10 °C was mixed, under nitrogen, with 300 mg of ammonium carbonate. After treatment with a small amount of acid-washed decolorizing carbon, the solution was filtered, saturated with cold acetone, and cooled in ice. The ammonium salt of I crystallized in nearly colorless needles. The product was recrystallized three times from aqueous acetone, and dried for 12 hr at 25 °C/0.1 mm; wt, 150 mg; mp 170 to 172 °C dec,

*Anal.* Calcd, for C_6_H_11_NO_6_·H_2_O: C, 34.1; H, 6.2; N, 6.6. Found: C, 34.1; H, 6.0; N, 6.6.

#### d. Catalytic Hydrogenation of I, and Separation cf Cyclitols

A mixture of 2 g of I, 150 ml of water, 50 ml of ethanol, and about 5 g of freshly prepared Raney nickel T–4 [22] was treated with hydrogen at 1350 psi, and the temperature was gradually raised to 100 °C. After 6 hr, the pressure was released, the mixture was filtered, and the filtrate was concentrated under reduced pressure to about half volume. The solution was treated with decolorizing carbon, refiltered, concentrated to about 10 ml, treated with acetone to incipient turbidity, and kept in a refrigerator for 18 hr. The resulting crystals were separated and dried; wt, 1.8 g.

A 500-mg sample of the cyclitol mixture was dissolved, with slight heating, in a mixture of 8 ml of acetic anhydride and 2 ml of 100-percent phosphoric acid, kept 30 min, and poured into ice water; the resulting crystals were collected on a filter, washed with water, and dried; wt, 0.8 g. A 300-mg sample of the crude product was extracted with 50 ml of boiling absolute ethanol, and the extract was concentrated, giving a crystalline product which was recrystallized several times from ethanol; mp 214 to 215 °C.

The ethanol-insoluble residue was recrystallized from 2:1 ethanol—acetic acid, to give colorless crystals, mp 300 to 301 °C. The melting points and infrared spectra agreed with those of the authentic hexaacetates of *myo*-inositol and *scyllo*-inositol, respectively.

#### e. Acylation

##### (1) Benzoylation

A mixture of I (0.5 g), 3 ml of benzoyl chloride, and 3 g of fused zinc chloride, in a test tube protected by a drying-tube, was heated at 110° for 2 hr and allowed to cool to room temperature. It was then diluted with 8 ml of absolute ethanol and kept in an ice bath for 2 hr. The resulting crystals (0.2 g) were separated and were recrystallized by suspending them in 40 ml of stirred, hot ethanol, gradually adding nitromethane until most of the product had dissolved, filtering, and cooling; mp 234 to 236 °C.

*Anal.* Calcd. for C_41_H_28_O_11_: C, 70.7: H, 4.1. Found: C, 70.4; H, 4.0.

##### (2) Acetylation without isomerization

Acetic anhydride (10 ml, containing 5 drops of perchloric acid) was added in small portions to a suspension of 1 g of I in 40 ml of ethyl acetate in a bath at 65 °C [23]. After the crystals had dissolved, the solution was kept at room temperature for 30 min, and poured into ice water. The mixture was extracted with chloroform, and the extract was successively washed with 5-percent aqueous sodium bicarbonate and water, dried with sodium sulfate, and concentrated to a sirup which was diluted with 8 ml of absolute ethanol to give colorless, glistening plates; these were recrystallized from 15 ml of hot ethanol; wt 1.5 g. The product melted at 111 to 113 °C, solidified above 113°, and again melted at 134 to 136 °C. The higher-melting form was converted to the lower-melting by recrystallization from ethanol ; the lower-melting was converted to the higher-melting by recrystallization from benzene.

*Anal.* Caled, for C_16_H_18_O_11_: C, 49.8; H, 4.7: CH_3_CO, 55.7. Found, for the product of mp 111 to 113 °C; C, 49.8; H, 4.7; CH_3_CO, 55.3. Found, for the product of mp 134 to 136 °C; C, 49.7; H, 4.8.

The analyses and infrared spectra of the two pentaacetates showed that the two materials are different crystal forms of the same substance.

A solution of the lower-melting form in methanol containing a few drops of concentrated hydrochloric acid, kept at room temperature for 2 hr and in a refrigerator for 3 days, deposited crystals of I. Hence, the compound is dl-*xylo*-pentaacetoxy-2-cyclohexen-1-one.

The pentaacetate was also obtained by acetylation of I with (a) acetic anhydride and zinc chloride, (b) acetic anhydride and 100-percent phosphoric acid, and (c) isopropenyl acetate and *p*-toluenesulfonic acid. Acetylation of I with acetic anhydride and sulfuric acid, however, gave a different pentaacetate, described below.

##### (3) Acetylation with isomerization

I (1 g) was added to a stirred mixture of 19 ml of acetic anhydride and 1 ml of concentrated sulfuric acid at 50 °C. After the crystals had dissolved (about 5 min), the mixture was cooled, kept at 20 °C for 1 hr, and poured into a mixture of ice and water. The mixture was extracted with chloroform, and the extract was washed, dried, and evaporated. The residue was recrystallized from absolute ethanol; yield, 1.2 g; mp 150 to 152 °C. By repeated recrystallization from ethanol, the melting point was raised to 154 to 155 °C.

*Anal.* Calcd, for C_16_H_18_O_11_: C, 49.8; H, 4.7; CH_3_CO, 55.7. Found: C, 49.6; H, 4.6; CH_3_CO, 56.1.

When this acetate was hydrolyzed by the method in the preceding section, it failed to yield the parent compound (I).

#### f. Aromatization

##### (1) Simultaneous acetylation and aromatization

I (1 g) was added in small portions to a hot, stirred mixture of 25 ml of acetic anhydride and 5 g of fused sodium acetate. After 15 min, the mixture was cooled, kept for 1 hr at room temperature, and poured into a mixture of ice and water, from which crude pentaacetoxybenzene crystallized; wt, 1.9 g. A solution of this in a hot mixture of 180 ml of absolute ethanol and 20 ml of glacial acetic acid was treated with decolorizing carbon, filtered, and kept for 18 hr in a refrigerator. The resulting crystals were separated and dried; wt, 1.5 g: mp 165 to 166 °C, in agreement with the value reported for pentaacetoxybenzene [14]. By recrystallization from hot, glacial acetic acid, the melting point was raised to 166 to 168 °C. The once-recrystallized material was used for preparing pentahydroxybenzene (section 5.3).

##### (2) Aromatization of acetates

For each of the three pentaacetates described in section 5.2e, a 250-mg sample was dissolved in a mixture of 10 ml of pyridine and 5 ml of acetic anhydride. The solution was heated to boiling, kept at room temperature for 2 hr, and poured into ice water. The product (approximately 200 mg) was separated and recrystallized from ethanol, or from a mixture of ethanol and acetic acid. Each of the products melted at 169 to 171 °C and showed an infrared spectrum identical with that of the product derived from I by simultaneous acetylation and aromatization.

### 5.3. Pentahydroxybenzene

A mixture of 1 g of pentaacetoxybenzene, 50 ml of methanol, and 5 ml of concentrated hydrochloric acid was placed in a 200-ml flask equipped with a reflux condenser. Under a stream of nitrogen, the mixture was gently boiled for 15 min; the light-yellow solution was then filtered, and concentrated under reduced pressure (40 °C). The resulting paleyellow crystals were separated by filtration, washed with 25 ml of cold ether, and dried in a vacuum desiccator; wt, 325 mg. For recrystallization, 100 mg was dissolved in 10 ml of boiling absolute ethanol; the solution was concentrated to about 2 ml, and pentane (about 2 ml) was added to incipient turbidity. The slightly pinkish crystals that separated on cooling were collected by filtration, washed with pentane, and dried; wt, 60 mg. The substance does not melt, but decomposes slowly at 255 to 265 °C. The analysis of the product did not correspond exactly with that of anhydrous pentahydroxybenzene, but was sufficiently close to show that the material was, indeed, pentahydroxybenzene.

*Anal.* Calcd, for C_6_H_6_O_5_: C, 45.6; H, 3.8. Found: C, 44.4; H, 3.8.

Freshly prepared methanolic solutions of pentahydroxybenzene are usually slightly yellow; they turn deep red on exposure to air. Ferric chloride solution imparts a pink color that rapidly deepens to magenta. Pentahydroxybenzene is sensitive to oxidation; even acid-washed decolorizing carbon causes a solution of the compound to turn deep red. At room temperature, the substance reduces Benedict solution and also silver nitrate, the latter almost instantaneously.

### 5.4. *xylo*-Trihydroxycyclohexane-1,2,3,-trione (II)

#### a. Preparation and Properties

A solution of I (1 g) in 10 ml of stirred, concentrated nitric acid was heated for 5 min on a steam bath, at which point II began to crystallize. The reaction mixture was cooled in ice, diluted with 25 ml of cold methanol, and kept 5 min. The resulting crystals were separated on a fritted-glass filter and washed with methanol; the filtrate was discarded.^10^ The fairly pure product began to darken at 200 °C and decomposed, without melting, at 220 to 225 °C.^11^

The compound may be recrystallized in low yield from hot water. II (1.5 g) was dissolved in 125 ml of hot water, and the solution was decolorized, filtered, and concentrated under reduced pressure to a sirup which was diluted with 50-percent aqueous methanol and kept at room temperature for 3 days. The resulting crystals were collected by filtration, washed with methanol, and dried; yield, 0.25 g. The infrared spectrum was unchanged by recrystallization. After being dried for 4 hr at 25 °C/0.1 mm, the compound was analyzed.

*Anal.* Calcd, for C_6_H_6_O_6_+1.5H_2_O: C, 35.8; H, 4.5. Found: C, 35.8: H, 4.6.

After being dried 1 hr at 110 °C/0.1 mm, it was reanalyzed. Calcd, for C_6_H_6_O_6_·H_2_O: C, 37.5; H, 4.2. Found: C, 38.0; H, 4.2.

A warm solution of II reduced Benedict solution instantaneously. On being heated, an aqueous solution of II decomposes slowly, giving oxalic acid as one of the decomposition products. An aqueous solution, treated at 60 °C, in air, with a solution of ammonium carbonate, changes color to deep yellow and finally deposits dark-blue needles of ammonium rhodizonate [17].

#### b. Simultaneous Acetylation and Aromatization

II (500 mg) was added to a hot, stirred mixture of 25 ml of acetic anhydride and 5 g of anhydrous sodium acetate. After 5 min, the mixture was cooled and poured into 100 ml of ice water. The solid that separated was collected by filtration, washed with water, and recrystallized from 15 ml of boiling acetic acid containing decolorizing carbon. The suspension was filtered and the filtrate cooled; the resulting crystals were separated, washed with acetic acid, and dried over sodium hydroxide in a vacuum desiccator; wt, 0.3 g; mp 203 to 205 °C. The product was identical, in melting point and infrared spectrum, with an authentic sample of hexaacetoxybenzene [24].

On hydrolysis with warm, 85-percent phosphoric acid, the acetate yielded needlelike crystals identical with those of hexahydroxybenzene [17].

#### c. Bis(phenylhydrazone)

II (300 mg) was dissolved in 75 ml of 7.5-percent aqueous acetic acid by warming slightly. To the stirred solution were added 50 g of crushed ice and 5 ml of phenylhydrazine. After 1 hr, the resulting red crystals were separated by filtration, washed with water, and dried; wt, 300 mg. The crude product was twice recrystallized from boiling methanol; dark-red needles, mp 184 to 186 °C (dec). Purified on a column of neutral alumina by elution with a 4:1 mixture of benzene and methanol, it melted at 186 to 187 °C.

The same bis(phenylhydrazone) was prepared from I as follows: A solution of 5 ml of phenylhydrazine in 12 ml of 50-percent aqueous acetic acid was added to a stirred solution of I (1.2 g) in 50 ml of water at 70 °C; the deep-red bis(phenylhydrazone) of II crystallized almost immediately. After 30 min, it was separated, washed with cold water, and dried wt; 1.2 g.

*Anal.* Calcd, for C_18_H_18_N_4_O_4_: C, 61.0; H, 5.1; N, 15.8. Found: C, 61.3; H, 5.0; N, 15.9.

### 5.5. Spectrophotometric Measurements

#### a. Apparatus and Techniques

The infrared spectra were recorded for the freshly prepared crystalline compounds, in Nujol mulls and in potassium chloride pellets, with a Perkin-Elmer Infracord Model 137 (double beam) spectrophotometer equipped with a prism of sodium chloride for the 2- to 15-*μ* range.

The ultraviolet spectra were determined with a Beckman DK–2 spectrophotometer having matched 1-cm quartz cells, with the relevant solvent as the reference standard. Molecular extinction coefficients are reported for those compounds for which the intensity of absorption did not change appreciably with time.

#### b. Discussion of Spectra

**Figure 6:**
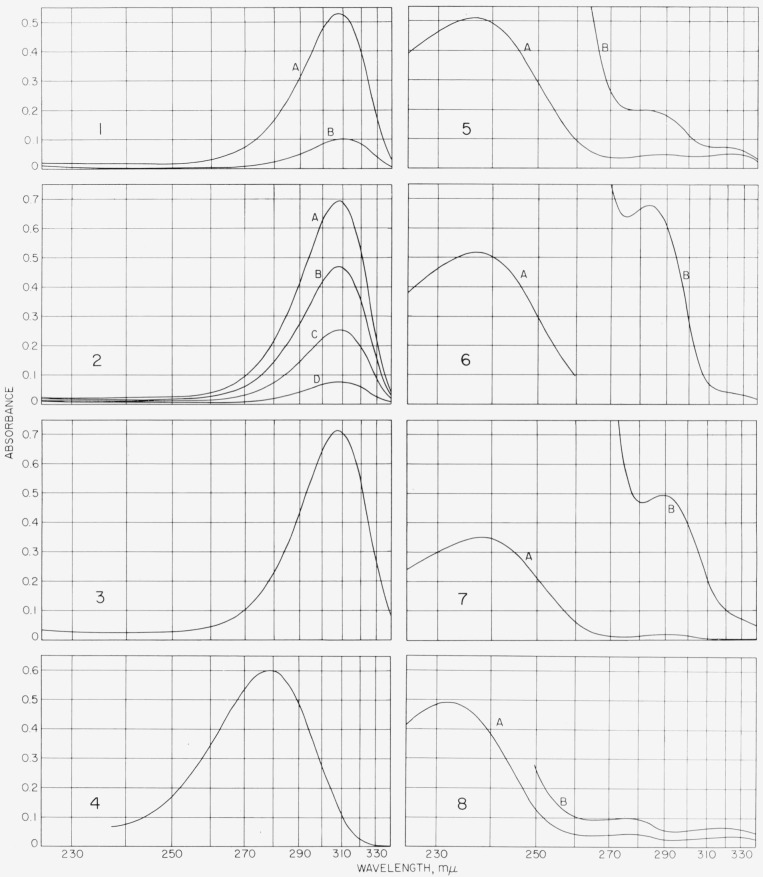

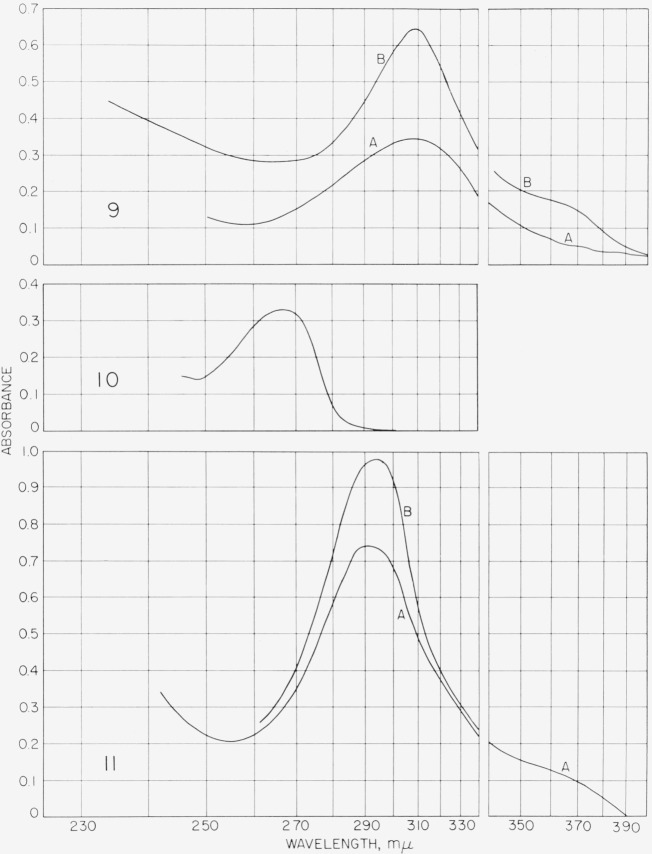
Ultraviolet spectrograms 1. dl-*xylo*-4, 5,6-Trihydroxycyclohexenediolic acid (I) in water, 3.6×10^−5^*M*, λ_max_ near 309m*μ*. A, 3 min after dissolution; B, 25 min after dissolution; 2. potassium dl-*xylo*-4,5,6-trihydroxycyclohexenediolate monohydrate in water, 3.9×10^−5^*M*, λ_max_ near 308m*μ*. A to D, respectively, 10, 16, 22, and 28 min after dissoultion; 3. ammonium dl-*xylo*-4,5,6-trihydroxycyclohexenediolate monohydrate in water, 7.4×10^−5^*M*, 15 min after dissolution, λ_max_ near 307m*μ*; 4. dl-*xylo*-4,5,6-trihydroxycyclohexenediolic acid (1) in 5-percent aqueous acetic acid, 4.8×10^−5^*M*, λ_max_ near 279m*μ*, *ϵ~*12,500; 5. dl-*xylo*-pentaacetoxy-2-cyclohexene-1-one, mp 111 to 113 °C (see sec. 5.2 e2) in methanol. A, 4.3×10^−5^*M*, λ_max_ near 237m*μ*, *ϵ~*12,400; B, 3.1×10^−4^*M*, λ_max_ near 283m*μ*, *ϵ~*700; 6. dl-*xylo*-pentaacetoxy-2-cyclohexene-1-one, mp 134 to 136 °C (see sec. 5.2 e2) in methanol. A, 4.3×10^−5^ *M*, λ_max_ near 237m*μ*, *ϵ~*12,400; B, 1×10^−3^*M*, λ_max_ near 283m*μ*, e~700; 7. pentaacetoxy-2-cyclohexene-l-one, mp 154 to 155 °C (see sec. 5.2 e3) in methanol. A, 20.×10^−5^M, X_max_near 238mμ, *ϵ~*17,500; B, 2.0×10^−3^M, λ_max_near 288m*μ*, *ϵ~*250; 8. dl-*xylo*-pentabenzoyloxy-2-cyclohexene-1-one; (see sec. 5.2 el) in methanol. A, 8.3×10^−6^*M*, λ_max_ near 232m*μ*; B, 1.65×10^−5^*M*, λ_max_ near 275m*μ*. 9. *xylo*-4,5,6-trihydroxycyclohexane-l,2,3-trione (II). A, in 5-percent aqueous acetic acid, 4.5×10^−3^*M*, 5 min after dissolution, λ_max_ near 306m*μ*. B, in warm water 9.1×10^−3^*M*, 10 min after dissolution, λ_max_ near 309m*μ*; 10. pentaacetoxybenzene (see sec. 5.2 f1) in glacial acetic acid, 4.7×10^−4^ *M*, λ_max_ near 267m*μ*, *ϵ*~580; 11. pentahydroxybenzene in methanol, 1.4×10^−4^*M*. A, 3 min after dissolution, λ_max_ near 292; B, 9 min after dissolution λ_max_ near 294m*μ*.

The ultraviolet absorption spectrum of the *enediolic acid I* varies with the solvent and the hydrogenion concentration. A freshly prepared, dilute aqueous solution of I exhibits a rapidly changing R-band ([25], p. 111; [26], p. 204) at λ_max_ near 309 m*μ*, almost identical with an absorption band at λ_max_ 307 m*μ* shown by the *potassium and the ammonium salt of I* (fig. 6, *1* to *3*). Hence, in dilute aqueous solution, I is present largely as the anion. The free acid and its potassium and ammonium salts are unstable in aqueous solution (in the presence of light and air), as evidenced by the rapid decrease in intensity of the absorption maxima.

The characteristic absorption of an aqueous solution (3.7×10^−5^*M*) of the potassium salt disappears in about 30 min at room temperature.

In acid solution (5-percent aqueous acetic acid or 2.5-*N* hydrochloric acid), the maximum of I shifts to shorter wavelengths, to provide a relatively stable band at λ_max_ near 279 m*μ* (fig. 6, 4). The presence of an acid stronger than I suppresses ionization and favors the nonionized form of I over the anion. Table 1 gives ultraviolet absorption maxima for several enediolic acids, including compound I. In each instance, the maximum for the nonionized form is at a shorter wavelength than that for the ionized form.

**Figure 7:**
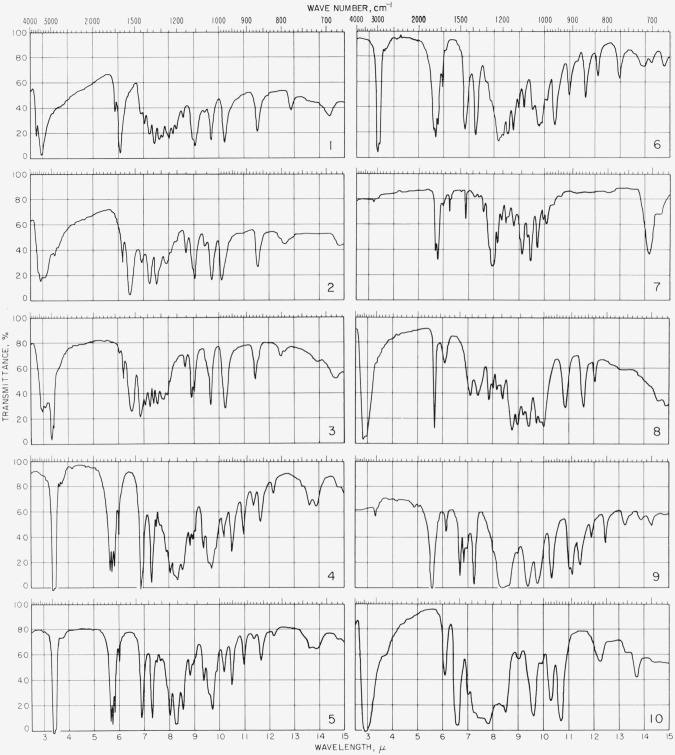
Infrared spectrograms of compounds in Nujol mulls and in potassium chloride pellets 1, dl-*xylo*-4,5,6-Trihydroxycyclohexenediolic acid (I) in potassium chloride pellet; 2, potassium salt of I in potassium chloride pellet; 3, ammonium salt of I in Nujol mull; 4, pentaacetate of I (mp 111–113 °C) in Nujol mull; *5*, pentaacetate of I (mp 134–136 °C) in Nujol mull; 6, isomeric pentaacetate of I (mp 154–155 C) in Nujol mull; 7, pentabenzoate of I in potassium chloride pellet; 8, *xylo*-4,5,6-trihydroxycyclohexane-l,2,3-trione in potassium chloride pellet; 9, pentaacetoxybenzene in potassium chloride pellet; 10, pentahydroxybenzene in potassium chloride pellet.

The *pentaacetate and pentabenzoate of I* show a K-band near 237 m*μ* and 232 m*μ*, respectively (fig. 6, *5, 6*, and *7*). It is of interest to compare these observed maxima with values calculated for the K-bands of *α*,*β*-unsaturated ketones by the empirical substitution rules of Woodward [27] and the Fiesers ([28], p. 19).^12^ If it is assumed that the effect of an acyloxy group is the same as that of an alkyl substituent (10 m*μ*), then, starting with methyl vinyl ketone (3-buten-2-one) (λ_max_ 215 m*μ*), the calculated absorption maximum for the pentaacetate and pentabenzoate of I should be 245 m*μ*. Although the agreement with the observed values is not particularly good, it seems probable that the effect of the acyloxy substituent is not 10 m*μ*, but of the order of 5 m*μ* ([25], p. 99; [29]). Use of the latter value gives a calculated absorption maximum of 235 m*μ*. The difference in the observed maxima for the pentaacetate and pentabenzoate may arise from steric effects ([30], p. 66).

Additional information concerning the structure of I and its derivatives can be obtained from their infrared spectra. Because of the low solubility of I in carbon tetrachloride, dilution studies to elucidate the type of hydrogen bonding present were not feasible. Either in potassium chloride pellets or Nujol mulls, with careful exclusion of moisture, I showed bands at 2.74 and 2.96 *μ* that can be attributed to hydroxyl groups (fig. 7, *1*). The spectrum also showed a moderately strong absorption band at 5.88 *μ* (C=O) and a strong band at 6.08 *μ* (*α*,*β*-unsaturated ketone).

The absence of a keto C=O band in the infrared spectrum of the potassium salt of I (fig. 7, *2*) is attributed to the fact that, in the anion, the carbonyl group is part of a resonance system involving C1 and C3, as shown in figure 1. A band at 6.45*μ* is assumed to arise from the anion of I, and is in fair agreement with a band at 6.35*μ* reported ([31], p. 68) for the anion of 5,5-dimethyl-l,3-cyclohexanedione (dimedone). An absorption band at 6.15*μ* is due, at least in part, to water ([32], p. 430; [33], p. 150; [34]).

The spectrum of the ammonium salt of I (tig. 7, *3*), like that of the potassium salt, fails to show a keto C=0 band; it shows bands at 6.45 and 6.20*μ*, presumably characteristic of the anion and of water of crystallization, respectively.

The infrared spectra of the acetates of I, mp 111 to 113 °C, 134 to 136 °C, and 154 to 155 °C, are given in figure 7, Curves *4*, *5*, and *6*, respectively. They are similar in the region from 2.5 to *7.5μ*, showing (a) a doublet near 5.63 and 5.70*μ* attributed to the presence of both vinyl and saturated acetates, (b) a band near 5.81*μ* (*α*,*β*-unsaturated ester), and (c) a band near 6.02*μ* (*α*,*ß*-unsaturated ketone). In the “fingerprint” region, two bands are common to all three, 8.02*μ* (acetate) and 8.33*μ* (vinyl acetate) ([35], p. 182; [36], p. 64; [32], pp. 483,484). However, curve *6* differs markedly from curves *4* and *5* at 7.5 to 7.7, 8.9, 10.2, 11.4, and 13.0*μ*. Some samples of the crude acetate (mp 111 to 113°C and 134 to 135 °C) of I, showed a strong absorption band at 13.0 *μ*, as did the acetate of mp 154 to 155 °C; on further purification of the acetate of mp 111 to 113 °C, particularly by change of solvent, the band disappeared. It thus appears that small proportions of the isomer of mp 154 to 155 °C are formed, even in the preparation of the low-melting acetate with “mild” catalysts (see p. 288).

The infrared spectrum of the benzoate of I (fig. 7, *7*) shows the bands characteristic of an *α*,*β*-unsaturated ester: a doublet at 5.69*μ* (vinyl ester) and 5.78*μ* (C=0); and a band at 6.00*μ* (C=C). Other bands are present at 6.22 and 6.70μ (phenyl) 6.90*μ* (C—H asymmetric deformation); and 7.95*μ* (aromatic ester ([36], p. 64)).

The *triketone* (II), unlike the enediolic acid (I), has almost the same ultraviolet spectrum in water, aqueous acetic acid, and methanol; the intensity of absorption at the observed λ_max_ at 302 to 306 m*μ* decreases with time (fig. 6, *8*). The identity of the spectra in water and aqueous acetic acid indicates that this band can be attributed to nonenolized carbonyl groups. The observed maximum is in approximate agreement with the maxima (295 to 301 m*μ*) reported for two isolated carbonyl groups [37]. Aqueous solutions of II show a weak band at about 365 m*μ*, in addition to the band at 302 to 306 m*μ*.

If the aqueous solution of II is treated with sodium carbonate, the solution becomes deep yellow, presumably through successive enolization and *beta*-elimination reactions, as represented for the acetates of II in figure 4. Bands appear in the ultraviolet and visible spectra near 296, 368, 422 (sh), and 482 m*μ*. The band at 368 m*μ* can be assigned to the anion of an enolized keto group [38]. The bands at 296, 422, and 482 m*μ* are similar to those observed for tetrahydroxy-*p*-benzoquinone in methanol at 312, 441, and 482 m*μ*^13^ [39] and for *p*-benzoquinone at 296 and 435 m*μ*. The bands for *p*-benzoquinone have been ascribed to a conjugated keto group [40]. Absorption by quinones in the region of 410 to 440 m*μ* has been ascribed to an n-π* transition [41, 42, 43].

When the alkaline solution of II is acidified with acetic acid, a broad band is produced with λ_max_ at 269 to 275 m*μ*; this band corresponds to a benzenoid structure ([25], pp. 116–120), in agreement with the expected aromatization and formation of *hexahydroxybenzene.* The solution, on treatment with methanolic ferric chloride, gave the magenta color typical of the reaction of phenols. Thus, the spectra and chemical properties indicate that II, in alkaline solution, undergoes aromatization and complex oxidation-reduction reactions.

The infrared spectrum of II (fig. 7, *8*) shows a single band at 5.70 *μ*, that is, at a somewhat shorter wavelength than that to be expected for dicarbonyl compounds (5.78 to 5.85 *μ* [36], p. 62, [32], pp. 483484). A band at 6.12 *μ* can be attributed to water ([32], p. 430, [34], [33], p. 150).

The ultraviolet spectrum of pentaacetoxybenzene, either in glacial acetic acid or in methanol (fig. 6, *9*), shows a single band, λ_max_ 267 to 268 m*μ*, within the region characteristic of benzenoid compounds ([25], p. 116).

The infrared spectrum of pentaacetoxybenzene (fig. 7, *9*) is consistent with the aromatic structure. Bands were observed at: 2.31 *μ* (C—H aromatic stretching); 6.15 *μ* (olefinic bond); 6.70 *μ* (aromatic ring); and 11.18 and 11.48 *μ* (C—H out-of-plane deformation of the isolated hydrogen atom on a pentasubstituted benzene ring). The strong band at 11.95 *μ* is probably *also* associated with an isolated hydrogen atom on the benzene ring ([35], pp. 69–79, [32], p. 394, [31], pp. 26, 27).

The methyl groups of the acetoxy substituents are associated with absorption at: 6.85 *μ* (C—H asymmetric deformation); 7.28 *μ* (C—H symmetrical deformation); 13.22 and 13.85 *μ* (C—H skeletal vibration) [36], p. 57). Ester bands were observed at 5.60 *μ* (phenolic acetate), and 8.00 *μ* (sh) (acetate). A strong band at 8.50 *μ* is probably associated with the acetoxy substituent ([36], p. 64).

Freshly prepared pentahydroxybenzene, when dissolved in methanol, shows a band at λ_max_ near 292 m*μ* whose intensity increases with time (fig. 6,*10*). Pentahydroxybenzene is extremely sensitive to oxidation by air. A methanolic solution, exposed to air, quickly acquires a red color and shows absorption bands at 288, 358, and 490 m*μ*. In comparison, 2,3,5-trimethoxy-*p*-benzoquinone absorbs at 288 m*μ* in ethanol [44], and hydroquinone absorbs at 294 m*μ* ([25], p. 119).

The infrared spectrum of pentahydroxybenzene (fig. 7, *10*) is also consistent with an aromatic structure. It shows a band at 6.10 *μ* (C=C) and at 6.55 *μ* (substituted benzene ring [35], pp. 71, 72). Characteristic strong bands observed at 12.25 and 13.70 *μ* are indicative of C—H out-of-plane deformation and of the presence of one free hydrogen atom on the ring. However, a band was not observed in the region of 3.4 to 3.5 *μ*, characteristic of C—H stretching. Bands found at 9.62, 10.30, and 10.80 *μ* are apparently associated with pentasubstitution on the ring ([35], p. 79; [36], p. 59; [32], p. 394).

## Figures and Tables

**Table 1:** Ultraviolet absorption maxima of some enediolic acids

Compound	Approximate maxima		References
	In aq HCl	In aq NaOH	
			
dl-*xylo*-Trihydroxycyclohexanediolic acid	*mμ* 279	*mμ* 307	……
l-Ascorbic acid	242	300	[1]
Reductic acid	a(266)	293	[2]
Croconic acid	295	365	[3]
Triose reductone	a(271)	292	[2]

a In ethanol.

[1] P. Karrer, H. Salomon, R. Morf, and K. Schöpp, Biochem. Z. 258, 4 (1933).

[2] G. Hesse and F. Urbanek, Ann. 604, 47 (1957).

[3] K. Yamada, N. Mizuno, and Y. Hirata, Bull. Chem. Soc. Japan 31, 543 (1958).

## Footnotes

3 Figures in brackets indicate the literature references at the end of this paper.

4 The term “enediolic acid” is used here for *α,β*-imsaturated, dihydroxy aldehydes and ketones having the structure . The compounds belong to the group that have been classified by their chemical reactions as “aci-reductones” [6, p. 3]. The enediolic acids are related to the carboxylic acids in that they have a carbonyl group in conjugation with a hydroxyl group. Typical examples are
Enediolic derivatives of tetrahydrofuran are more conveniently named as ascorbic acids, for example:

5 After oxidizing (the d isomer of) dl-*epi*-inosose-2 with *Acetobacter suboxydans*, Magasanik and Chargaff [8] found that the oxidation product had “acidic properties … in agreement with the expected behavior of an enolized *β*-di-ketone.” This compound, which was not isolated, may have been the enediolic acid described here.

6 By treatment of *myo*-inositol with concentrated nitric acid according to Posternak [3, 20, 21], it is possible to obtain the same product (I) reported here, but in lower yield and more contaminated with byproducts.

7 When the mother liquor, after separation of the inosose, was neutralized with potassium bicarbonate instead of potassium carbonate, about 80 percent of the resulting product was the dipotassium salt of tetrahydoxy-*p*-benzoquinone. This compound can be oxidized in air to dipotassium rhodizonate [17].

8 A slurry of Darco 60 (Atlas Chemical Industries Inc., Wilmington, Del.) in 15-percent, aqueous hydrochloric acid was heated at 80 °C for 24 hr. The carbon was collected by filtration, washed with distilled water until free from chloride ion, and dried at 85 °C in a vacuum oven.

9 The colored potassium salt obtained here is adequate for preparing the free enediolic acid (sec. 5.2a). The preparation of the pure potassium salt, which is colorless, is described in section 5.2b.

10 Mixtures of nitric acid and methanol should not, of course, be concentrated with heating because of the danger of violent decomposition.

11 Compound II can also be obtained by oxidation of I with iodine, neutral potassium permanganate, or a cold solution of silver nitrate.

12 See summary by Jaffe and Orchin ([26], pp. 218, 219).

13 In addition, there is a strong band in the visible region, at 510 m*μ*.
